# NF-κB as potential target in the treatment of melanoma

**DOI:** 10.1186/1479-5876-10-53

**Published:** 2012-03-20

**Authors:** Gabriele Madonna, Claudio Dansky Ullman, Giusy Gentilcore, Giuseppe Palmieri, Paolo Antonio Ascierto

**Affiliations:** 1 Unit of Medical Oncology and Innovative Therapy, Istituto Nazionale Tumori Fondazione, G. Pascale, Napoli, Italy; 2 Cancer Therapy Evaluation Program, National Cancer Institute, Bethesda, USA; 3 Institute of Biomolecular Chemistry-CNR, Sassari, Italy

**Keywords:** NF-κB, Melanoma, NBD peptide, Bortezomib, Curcumin

## Abstract

The RAS/MAP kinase pathway has attracted attention because activating mutations of the BRAF serine/threonine kinase was described in over 50% of melanomas. Very recently, selective and potent BRAF inhibitors have been developed. Several other signal transduction pathways have been found to be constitutively active or mutated in other subsets of melanoma tumors that are potentially targetable with new agents. Among these, NFκB is another pathway that melanoma tumors use to achieve survival, proliferation and resistance to apoptosis. Inhibition of NF-κB activation appears to be a very promising option for anti-cancer therapies.

## Introduction

Melanoma is considered the most aggressive form of skin cancer, derived from activated or genetically altered epidermal melanocytes. In recent years, an alarming increase in incidence rates, higher than that observed for all other types of malignant tumors, has been registered [1].

Human malignant melanoma is a highly metastatic cancer that is markedly resistant to conventional therapy, with dacarbazine or temozolomide (TMZ); indeed, the best single agent activity presents a response rate of only 15-30% and a median duration of response of few months [2]. For its biological complexity and clinical difficulty of management, melanoma is considered a thorn in oncology's flesh. Recent achievements in the understanding of its molecular complexity and biology has resulted in important improvements in its therapeutic management. Still, further insights are needed.

Recent studies have focused on various molecular levels to identify key factors involved in the induction and progression of melanoma.

The RAS/MAP kinase pathway has attracted attention because activating mutations of the BRAF serine/threonine kinase has been detected in more than 50% of melanomas; in particular, the most common *BRAF *mutation (nearly, 90% of cases) is the T1799A point mutation in exon 15 within the kinase domain, in which a T → A transversion converts glutamic acid for valine at the 600 position of the amino acid sequence (*BRAF*^V600E^) and constitutively activates the protein. Oncogenic activation of BRAF may drive the positive control of cell cycle progression, cyclin-D1 mediated [3].

Other mutations occur in *NRAS *(present in 15% across all types of melanoma), *MEK1, MEK2 *as well as in *c-KIT*, encoding for an upstream growth factor receptor. The latter has been shown to be amplified or mutated in some cases of melanomas and in particular the prevalence is closer to 1% in the Caucasian population, specifically those that develop on body sites with little UV exposure. Activation of this tyrosine kinase results in the stimulation of the MAPK and PI3K-AKT pathways, producing both proliferative and survival advantages [4].

The study and the discovery of DNA mutations in melanoma provides new tools for controlling the disease: indeed, it has been possible to study and use drugs directed to the inactivation of proteins erroneously activated during melanoma pathogenesis [5].

Sorafenib was tested in melanoma as a BRAF inhibitor in combination with chemotherapy in both first and second line phase 3 trials, but failed to provide an improvement in outcome compared to chemotherapy alone [6,7]. Presumably, this was due to a suboptimal ability of Sorafenib to inhibit BRAF and lack of patient selection for BRAF mutations at the time the studies were conducted.

More selective and potent BRAF inhibitors have been developed. Vemurafenib is the first of a new generation of BRAF inhibitors that showed very promising activity in a phase 1 study, subsequently confirmed in phase 2 and 3 studies, among BRAF mutation positive patients. The phase III study comparing vemurafenib to dacarbazine showed a relative reduction of 63% in the risk of death and of 74% in the risk of either death or disease progression [8].

Another innovative approach was based on the use of monoclonal antibodies (Mabs) that specifically target CTLA-4 (cytotoxic T lymphocyte-associated antigen 4). CTLA-4 signaling switches off T-cell activation and induces immune tolerance. Inhibiting CTLA-4 increases and prolongs the antitumor T-cell response. Tremelimumab and ipilimumab were the most important anti-CTLA-4 Mabs used in the treatment of advanced melanoma. Although tremelimumab failed to improve outcome in the phase 3 setting [9], ipilimumab has been successful approved for the treatment of metastatic melanoma. Two phase 3 trials, one in the second line and another in first line, have shown improvement in survival with the use of this latter agent; durable responses have been shown with manageable toxicities [10].

Actually, these new therapies are under investigation for their combination with treatments aimed at blocking other key molecular pathways involved in the progression of melanoma [11].

Several other signal transduction pathways have been found to be constitutively active or mutated in other subsets of melanoma tumors that are potentially targetable with new agents. Among these, NFκB is another pathway that melanoma tumors use to achieve survival, proliferation, resistance to apoptosis and metastasis. In fact, it has been demonstrated that upregulation of the NF-κB levels is involved in both the progression of melanoma [12] and the increase of its metastatic potential [13].

Therefore, inhibition of NF-κB activation appears to be a promising option for anti-cancer therapies.

### NF-κB transcription factor: its role in cellular life

NF-κB is a complex protein that acts as a transcriptional factor and regulates the transcription of several genes involved in many critical pathways. It was identified many years ago as a regulator of B cell-specific gene expression; recently, studies have highlighted that it is a member of the structurally-related eukaryotic transcription factors family NF-κB/Rel that regulates the expression of many inducible genes involved in the control of a large number of normal cellular and organismal processes [14]. Specific NF-κB binding sites characterized by the consensus sequence: gggRNNYYCC (R = purine Y = pyrimidin), have been identified in promoters and enhancers of a great number of genes. This family is composed by related, evolutionarily conserved DNA-binding proteins consisting of p50, p52, RelA/p65, c-rel and RelB. The NF-κB proteins share an approximately 300 amino acid N-terminal domain called the Rel homology (RH) domain containing important sequences for binding DNA or inhibitor of NF-κB (I*κ*B) as well as sites of dimerization. However, they differ in their C-terminal domain in that RelA, RelB and c-Rel exhibit transactivating functions, while p100 and p105 contain inhibitory domains. In fact, NF-κB1/p105 and NF-κB2/p100 are the inactive precursors of the p50 and p52 proteins, respectively. In an unstimulated state, these proteins are localized into the cytoplasm. Proteolytic processing removes the C-terminal inhibitory domains, allowing the resulting proteins to enter the nucleus. p50 and p52 usually form homodimers or heterodimers with one of the three proteins that has a transactivation domain. NF-κB proteins are sequestered in the cytoplasmic compartment, associated with members of the I*k*B family (I*k*B*a*, I*k*B*b *and I*k*B*e*). Their activation is the result of a variety of different stimuli, including those activating some membrane receptors [B cell receptor (BCR), tumor necrosis factor receptors (TNFR)] and several extracellular stimuli (inflammatory cytokines, viral and bacterial infections, oxidative and DNA-damaging agents, UV light and osmotic shock) [15].

Activation of these signaling cascades is involved into phosphorylation of two critical serine residues in I*k*B proteins. Such a modification triggers their ubiquitination and destruction via proteasome degradation machinery. As a consequence, free NF-κB translocates to the nucleus and activates transcription in multiple genes involved in pathways controlling immune and inflammatory responses, developmental processes, cellular growth and apoptosis [16].

This event depends on activation of an upstream multimeric IKK complex, formed by two highly homologous kinases, IKKα and IKKβ, and one non-enzymatic protein, IKKγ or NEMO (NF-κB essential modulator) and a newly identified protein ELKS.

NEMO is a regulatory component, considered as indispensable for the formation of the IKK complex through a bound to the specific carboxyl-terminally conserved residues (termed NEMO binding domain; NBD) of both IKKα and IKKβ. The trimeric complex phosphorylates IκΒ and induces its proteasomal degradation via ubiquitination. NF-κB is then capable to migrate into the nucleus and activate the transcription of target genes. This classical pathway mostly involving p50:RelA and p50:c-Rel dimers strongly depends on IKKβ activity.

Activation of trimeric IKK complex is triggered by microbial and viral infections or proinflammatory stimuli and is responsible, under certain conditions, for inhibition of apoptosis as well as inflammatory and innate immune responses [17]. In addition to the classical pathway, a non-canonical alternative pathway exists, that does not depend on the interaction of IKKβ and NEMO but uniquely on the specific activation of a homodimer of IKKα, which is activated by the upstream kinase NIK and members of the TNF cytokine family (BAFF). This pathway leads to selective activation of NF-κB2/p100 precursor protein that resides in the cytoplasm; this event leads to the formation of a heterodimer with RelB that traslocates in the nucleus and activates the transcription of important genes involved in cell survival [18].

### Constitutive activation of NF-κB in melanoma

NF-κB has pleiotropic effects in the surrounding environment, but three are its essential roles.

It is involved into the proinflammatory response: a first line of defense against infectious diseases and cellular stress and comprises immune, inflammatory, and acute phase responses.

NF-κB is also a major anti-apoptopic factor, inducing the trascription of several anti-apoptotic proteins, such as Bcl-XL, tumor necrosis factor receptor-associated factor 1 and 2 (TRAF1 and TRAF2, respectively), and the inhibitor-of-apoptosis (IAP) protein 1 and 2 (c-IAP1 and c-IAP2, respectively). Therefore, NF-κB curbs the activities of the caspase family of enzymes, which are central in most apoptotic processes.

Finally, NF-κB promotes cell growth: activated NF-κB enhances the expression of cyclin D1 that is an important regulator of cell cycle progression.

Given the breadth of biological mechanisms associated with NF-κB, it is not surprising that NF-κB pathway is altered in several malignancies.

In melanoma cells, recent studies have highlighted that some components of NF-κB family, such as p50 and p65/RelA proteins, are overexpressed in the nuclei of dysplastic nevi and melanoma cells compared to those of normal nevi and healthy melanocytes, respectively [19].

These phenomena are more closely associated with an increased activity of IKK, resulting in more rapid degradation of I*κ*B, nuclear localization of NF-κB, and enhanced transactivating capacity of the NF-κB complex. In fact, the equilibrium between IκBα degradation and resynthesis has been found altered in Hs294T melanoma cells, with a shift of the process toward the first event (degradation) leading to constitutive nuclear translocation and activation of NF-κB [20].

Other data show that a hyperactivation of NF-κB can be also caused by an increased expression of other factors involved indirectly in NF-κB pathway. Recent studies on the gene expression profile of melanoma cells have shown an increased expression of Osteopontin (OPN) [21], a secreted glycophosphoprotein that induces NF-κB activation through enhancement of the IKK activity based on phosphorylation and degradation of IκBα [22]. Indeed, OPN induces Akt phosphorylation and, in turn, phosphorylated Akt binds to IKKα/β and activates IKK complex [22].

Mutational activation of BRAF, common in human melanomas, has been also associated with an enhanced IKK activity and a concomitant increase in the rate of IκBα ubiquitination and its subsequent degradation. This process overall entails a constitutive induction of NF-κB activity and an increased survival of melanoma cells [23]. Combination of these data with others reported in literature strongly suggests that the enhanced activation of NF-κB may be due to deregulations occurring in upstream signaling pathways such as RAS/RAF, PI3K/Akt and NIK [24]. In transformed cells, these alterations lead to a consequent increase in proliferation rates and resistance to apoptosis; moreover, activation of NF-κB may enhance expression of proinflammatory mediators, leading to acute inflammatory injury in multiple organs and development of dysfunction as well as cancer in several anatomical districts.

### Inhibitors of NF-κB as therapeutic option in melanoma

All findings presented above suggest that inhibition of NF-κB can be used as a strategy to effectively interfere with the pathogenesis and/or progression of melanoma. However, the multiplicity of actions of NF-κB could have the drawback that this type of therapy might entail disadvantageous effects: a reduction of the NF-κB activation could lead to an impairment of either the migration of natural killer (NK) cells or the concentration of the tumor infiltrating lymphocytes (TILS) and dendritic cells into the developing tumor. All these effects might reduce or abolish the immune response against the tumor. Recent studies with substances that inhibit the activity of NF-κB have hypothesized that these unwanted biological effects in tumor tissues may be apparently overcome by a much stronger enhancement of apoptosis into the tumor cells [25]. Several molecules targeting different parts of the NFκB pathway are here discussed.

### Bortezomib

Recent studies have shown that proteasome inhibitors represent a new class of anticancer agents by inhibiting degradation of cell cycle regulatory proteins, such as cyclin-dependent kinase inhibitors and IκB protein. Since NF-κB activity is regulated by phosphorylation and proteasome-dependent degradation of its inhibitor IκB, the use of a proteasome inhibitor may represent an effective approach into the treatment of melanoma with constitutive activation of NF-κB.

Bortezomib (Velcade, previously known as PS-341), a proteasome inhibitor, was one of the first compounds used to inhibit the function of NF-κB. Bortezomib is an anticancer agent that is not subject to classic MDR-dependent inactivation; it has a wide spectrum of actions in hematologic and solid tumors, including multiple myeloma as well as lung, breast, prostate, pancreatic, and head-neck carcinomas [26]. In melanoma, Bortezomib inhibits the cell growth *in vitro *, even at low drug concentrations (range of 0.1-10 nM) [27]. This compound was subsequently tested in combination with temozolomide, a conventional chemotherapeutic agent, resulting in a more prominent inhibition of the melanoma cell proliferation [27]. This compound was finally tested *in vivo *in mouse models, either alone or in combination with temozolomide. A markedly reduction of the tumor growth was observed when bortezomib was administered as single agent; more strikingly, animals receiving bortezomib in combination with temozolomide achieved complete remission of palpable tumors after only 30 days of therapy [27].

Recently, a phase I safety/dose-finding study combining Bortezomib with temozolomide in 17 patients with advanced metastatic melanoma was reported [28]. A phase 2 combination dose of Bortezomib (1.3 mg/m2 on d1, 4, 8, and 11 q 21 days) and TMZ (75 mg/m2 daily for 6 out of 9 weeks) was established. Side effects included grade 3 and 4 fatigue, nausea and vomiting, diarrhea, neuropathy, and rash [28].

The efficacy of Bortezomib was recently explored in another 2-stage phase 2 clinical trial in combination with other chemotherapeutic agents, including paclitaxel (PAC) and carboplatin (CBDCA) in patients with advanced solid tumors. This study has shown limited clinical advantage and significant toxicity (severe hematologic toxicities, such as neutropenia and leukopenia; severe pulmonary embolism); for these reasons this trial did not proceed to second-stage accrual [29].

### Curcumin

Another compound that has been shown to exert numerous effects - primarily antioxidant, anti-inflammatory, antiproliferative, and pro-apoptotic activities against a variety of tumors *in vitro *is curcumin (diferuloylmethane), a polyphenol with demonstrated anticancer properties, extracted from the rhizome of the plant *Curcuma longa *which represents a yellow pigment used as a food additive isolated from the spice turmeric (curry powder).

The antiproliferative and proapoptotic effects are associated with the ability of curcumin to downregulate the levels of some transcription factors (such as NF-κB, AP-1, Egr-1), growth factors receptors (such as epidermal growth factor receptor [EGF] and human EGF receptor 2), other signaling proteins (such as COX2, LOX, NOS, MMP-9, uPA, TNF, chemokines, cell surface adhesion molecules and cyclin D) and other tyrosine kinases and serine/threonine kinases protein [30]. Among others, one of the major anticancer effects of curcumin is due to its capability of triggering apoptosis in tumour cells through modulation of the NF-κB and the PI3K/Akt mediated signalling pathways [31]. These evidence suggest that curcumin may modulate pathways which contribute to chronic inflammation underlying tumorigenesis or pathways directly involved in tumorigenesis.

For these properties, curcumin was used in experiments based on melanoma cells as *in vitro *and *in vivo *models. Using a MTT proliferation assay, melanoma cell lines treated with curcumin for 72-96 hours were significantly inhibited in cell growth and survival, in an irreversible manner [32]. Furthermore, curcumin was found to induce a dose-dependent increase in apoptosis among human melanoma cell lines [32].

In other studies, curcumin was demonstrated to be cytotoxic *in vitro *for B16-R melanoma cells resistant to treatment with doxorubicin in monolayer cultures; such a cytotoxic effect has been related to the induction of programmed cell death [33]. In 3-D cultures of B16-R cells (called spheroids) - that are more resembling *in vivo *solid tumors, higher doses of the compound are needed in order to achieve the same antiproliferative effects since the spheroids cells are more resistant to the cytotoxicity induced by curcumin. To test curcumin's antitumoral effects *in vivo*, a first group of mice carrying melanoma lesions were pretreated with an immune therapy that contained proteins derived from the melanoma cells, while another group was treated with curcumin, and a third group received both treatments. Despite each treatment alone showed a poor response, the combination of them resulted in a substantial inhibition of tumor growth and a considerable improvement of the immune reaction against tumor lesions [33]. When survival was evaluated, the simultaneous administration of the immune therapy and curcumin was found to markedly improve median survival (which has been estimated to be increased by roughly 83 percent as compared to untreated mice using a combined treatment) [33].

In conclusion, curcumin could be used as an adjuvant compound in the treatment of melanoma. The importance of curcumin also lies in the fact that this drug appears to reduce the metastatic potential of melanoma, which is the main cause of death in patients. Treatment of highly metastatic murine melanoma B16F10 cells with curcumin (15 μM) for 15 days significantly inhibited matrixmetalloproteinase-2 (MMP-2) activity, which has been described to promote cell motility and metastasis formation, thus increasing the metastatic potential [34]. Expression of MMP-2 did not return to control levels even after several weeks form drug withdrawal [34]. Reduction of MMP-2 activity has been therefore indicated as an important mechanism by which curcumin exert its anti-metastatic property [34]. Recently, a new curcumin-related biphenyl structure (a,ß-unsaturated ketone D6) with more effective and selective antiproliferative and proapoptotic activity on both melanoma cell lines and in vivo melanomas has been described [35].

So far, no apparent side effects have been reported in literature when using curcumin-based treatments. Increased body temperature represents the most common side effect during treatment with high doses of curcumin. However, this issue remains controversial; a study performed at the University of Michigan showed no curcumin toxicity at doses up to 6 grams [36], whereas a recent trial based on curcumin given in combination with gemcitabine in patients with advanced pancreatic cancer reported some important side effects, such as intractable abdominal fullness or pain and other abdominal complaints [37].

### Selective inhibitors of NF-κB pathway

The above-mentioned treatments include substances that block, in addition to the NF-κB pathway, other molecular pathways, because their action is not directed solely and specifically to NF-κB but to a broad spectrum of proteins. The nonspecific action of these drugs does not allow to fully understand the role of NF-κB in cancer and its real value as a therapeutic target. On this regard, the attention has recently shifted to possible agents that could more selectively target the NF-κB pathway.

A compound that more directly targets the NF-κB pathway is BMS-345541 (4(2'-aminoethyl) amino-1,8-dimethylimidazo(1,2-a)quinoxaline). It was identified as a selective inhibitor of the catalytic subunit of IKK that binds to an allosteric site of the enzyme [38]. The activity of this compound was tested in vitro on three melanoma cell lines that have high levels of constitutively active IKK. The treatment induces a concentration-dependent inhibition of melanoma cell proliferation and apoptotic features, such as obvious nuclear condensation and DNA fragmentation. Successively BMS-345541 was tested in melanoma-bearing mice observing a dose-dependent suppression of tumor growth with good tolerance [39].

Besides the BMS-345541, several compounds are actually at different stages of development. Among others, a significant inhibitory activity has been recently documented for the NBD (NEMO-binding domain) peptide [40]. This is a short peptide with high affinity to the IKK complex and it is fused with a sequence derived from the *Drosophila antennapedia *homeodomain that mediates membrane translocation. In more details, the NBD is a hexapeptide sequence (Leu-Asp-Trp-Ser-Trp-Leu) within the extreme carboxyl-terminal region of IKKα and IKKβ that binds an NH2-terminal α-helical region of NEMO [41]. The interaction between NEMO and IKKα/β is crucial for the activation of the IKK complex and the subsequent activation of NF-κB.

**Figure 1:**
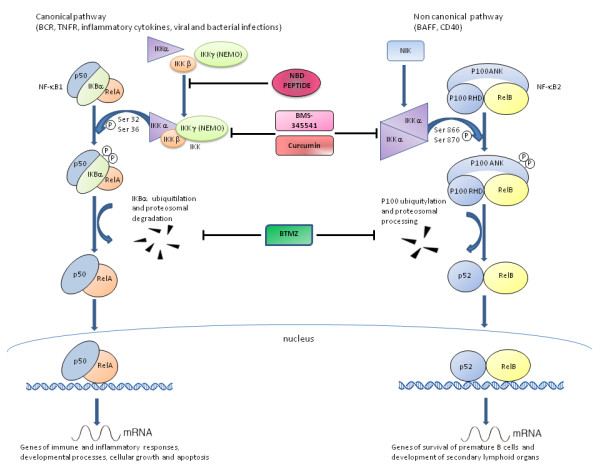
**Canonical and non canonical NF-kB pathways and related drugs inhibition**. The canonical pathway induces activation of IkB-bound NF-κB species (such as relA/p50 or cRel/p50) through activation of an upstream multimeric IKK complex, formed by IKKα, IKKβ, and IKKγ or NEMO; this activation results in the phosphorylation (P) of IkBα, its ubiquitylation (Ub) and subsequent degradation by the 26S proteasome. Release of the NF-κB complex allows it to relocate to the nucleus. The non canonical pathway depends on activation of IKKα dimers only. Four drugs that are used to block NF-κB pathways with different mechanism are represented. Bortezomib is a proteasome inhibitor that blocks the degradation of IkBα; curcumin and BMS-345541 inhibit IKK complex formation (both heterodimeric that omodimeric forms); NBD peptide targets the NBD-NEMO interaction. The first three compounds block both canonical and non canonical signaling, whereas NBD peptide blocks the canonical pathway only.

This last evidence has led to the hypothesis of using a sequence that mimics the NBD region in the treatment of pathologies in which NF-κB plays an important role. For this purpose, a cell-permeable synthetic NBD peptide was used in inflammatory pathologies, such as inflammatory arthritis and synovial inflammation with good results also confirmed in animal models of inflammation [42]. The molecular mechanism underlying the activity of the NBD peptide is here summarized. The NBD peptide inhibits the activation of NF-κB by targeting the NBD-NEMO interaction; when IKKα and IKKβ are bound to the NBD peptide, they cannot bind to NEMO and thus the formation and function of the IKK complex is blocked (Figure 1).

Recently, our group has conducted a study aimed at evaluating the efficacy of treatment with the NBD peptide in melanoma cells *in vitro*. It has been shown that A375 human melanoma cells, which present high constitutive levels of NF-κB, treated with different concentrations of synthetic NBD peptide, undergo a growth arrest in a dose-dependent manner; this effect is accompanied and strengthened by the impaired ability of NF-κB to bind to DNA [43]. It was also demonstrated that the reduction of cell proliferation induced by the NBD peptide is due to a significant increase of apoptosis, as shown by the presence of the cleaved form of poly-(ADP ribose)-polymerase (PARP-1) protein, considered one of the best markers of the apoptotic machinery [43]. All these effects were not detected using a control peptide having a mutation in its consensus sequence [43].

These investigations have been then extended to other nine melanoma cell lines in which the activity of NF-κB resulted strongly reduced following treatment with NBD (Gentilcore et al., unpublished observations). Although not all the nine cell lines have the same baseline expression of NF-κB, the NBD peptide was able to induce activation of the pro-apoptotic caspase-3 protein in the entire series of melanoma cell lines with a quite homogeneous distribution of the activation levels among the different cell lines (Gentilcore et al., unpublished observations.). Anyway, all these data demonstrate how NF-κB can be used as a specific target in the treatment of melanoma. Furthermore, it is worth extending the use of synthetic peptide NBD in *in vivo *studies based on animal models and, finally, in clinical trials among humans.

## Conclusions

NF-κB signaling is deregulated in many tumor types, resulting in aberrant expression and/or activation of NF-κB transcriptional complexes. Its role in the regulation of apoptosis, tumor angiogenesis and proliferation, as well as tumor cell invasion and metastasis indicates that this transcription factor may become a therapeutic target in the treatment of many human cancers, including melanoma, in which the levels of NF-κB is constitutively increased.

The existence of several NF-κB inhibitors may result in a significant improvement of the anti-tumor tools against melanoma, both *in vitro *and *in vivo*. More importantly, highly specific inhibitors of NF-κB may be used to minimize the pleiotropic effects of nonspecific inhibitors and to reduce toxicity *in vivo*. This field of research is new, with great potentiality, and worth being exploited in order to provide more and alternative options for the treatment of melanoma.

## Competing interests

Dr Paolo A. Ascierto participated to Advisory Board from Bristol Myers Squibb, MSD, Roche-Genentech, GSK, and received honoraria from Brystol Myers Squibb, MSD and Roche-Genentech. All remaining authors have declared no Competing Interest.

## Authors' contributions

GM performed data acquisition, data analysis, data interpretation, preparation of illustrations and drafted the manuscript; CDU revised the manuscript critically for important intellectual content and for the language; GG helped in the interpretation of data and in the drafting of the manuscript; GP collaborated in the drafting of the manuscript and gave an intellectual contribution; PAA conceived the study, drafted the manuscript and provided overall supervision in the project; all authors read and approved the final manuscript.
